# Fluorescence Liquid Biopsy for Cancer Detection Is Improved by Using Cationic Dendronized Hyperbranched Polymer

**DOI:** 10.3390/ijms22126501

**Published:** 2021-06-17

**Authors:** Violeta Morcuende-Ventura, Sonia Hermoso-Durán, Natalia Abian-Franco, Roberto Pazo-Cid, Jorge L. Ojeda, Sonia Vega, Oscar Sanchez-Gracia, Adrian Velazquez-Campoy, Teresa Sierra, Olga Abian

**Affiliations:** 1 Instituto de Nanociencia y Materiales de Aragón (INMA), Química Orgánica, Facultad de Ciencias, CSIC-Universidad de Zaragoza, Pedro Cerbuna 12, 50009 Zaragoza, Spain; violeta.m.v@csic.es; 2 Joint Units IQFR-CSIC-BIFI and GBsC-CSIC-BIFI, Institute of Biocomputation and Physics of Complex Systems (BIFI), Universidad de Zaragoza, 50018 Zaragoza, Spain; shermosod@gmail.com (S.H.-D.), svega@bifi.es (S.V.), adrianvc@unizar.es (A.V.-C.); 3 Instituto de Investigación Sanitaria Aragón (IIS Aragón), 50009 Zaragoza, Spain; 4 Hospital Reina Sofía, Carr. Tarazona, Km. 4, 31500 Tudela, Spain; nabianfranco@gmail.com; 5 Hospital Universitario Miguel Servet (HUMS), Paseo Isabel la Católica, 1-3, 50009 Zaragoza, Spain; rpazo@salud.aragon.es; 6 Department of Statistical Methods, Universidad de Zaragoza, 50009 Zaragoza, Spain; jojedacabrera@gmail.com; 7 SOTER BioAnalytics, Enrique Val, 50011 Zaragoza, Spain; oscar.sanchez.gracia@gmail.com; 8 Departamento de Bioquímica y Biología Molecular y Celular, Universidad de Zaragoza, 50009 Zaragoza, Spain; 9 Centro de Investigación Biomédica en Red en el Área Temática de Enfermedades Hepáticas y Digestivas (CIBERehd), 28029 Madrid, Spain; 10 Fundación ARAID, Gobierno de Aragón, 50018 Zaragoza, Spain; 11 Instituto Aragonés de Ciencias de la Salud (IACS), 50009 Zaragoza, Spain

**Keywords:** pancreatic ductal adenocarcinoma **(**PDAC), ovarian cancer, fluorescence spectroscopy, dendrimers, liquid biopsy, diagnosis, generalized linear models

## Abstract

(1) Background: Biophysical techniques applied to serum samples characterization could promote the development of new diagnostic tools. Fluorescence spectroscopy has been previously applied to biological samples from cancer patients and differences from healthy individuals were observed. Dendronized hyperbranched polymers (DHP) based on bis(hydroxymethyl)propionic acid (bis-MPA) were developed in our group and their potential biomedical applications explored. (2) Methods: A total of 94 serum samples from diagnosed cancer patients and healthy individuals were studied (20 pancreatic ductal adenocarcinoma, 25 blood donor, 24 ovarian cancer, and 25 benign ovarian cyst samples). (3) Results: Fluorescence spectra of serum samples (fluorescence liquid biopsy, FLB) in the presence and the absence of DHP-bMPA were recorded and two parameters from the signal curves obtained. A secondary parameter, the fluorescence spectrum score (FS_score_), was calculated, and the diagnostic model assessed. For pancreatic ductal adenocarcinoma (PDAC) and ovarian cancer, the classification performance was improved when including DHP-bMPA, achieving high values of statistical sensitivity and specificity (over 85% for both pathologies). (4) Conclusions: We have applied FLB as a quick, simple, and minimally invasive promising technique in cancer diagnosis. The classification performance of the diagnostic method was further improved by using DHP-bMPA, which interacted differentially with serum samples from healthy and diseased subjects. These preliminary results set the basis for a larger study and move FLB closer to its clinical application, providing useful information for the oncologist during patient diagnosis.

## 1. Introduction

In recent years, cancer has emerged as the second leading cause of death worldwide, accounting for almost 10 million deaths per year, according to estimates from World Health Organization (WHO) [1]. Early detection and diagnosis of cancer is the best means for reducing the associated mortality and morbidity associated with it because long term survival rates drop significantly once metastasis has occurred [1]. Developing novel non-invasive (or minimally invasive) strategies for early-stage cancer diagnosis with sensitivity may contribute considerably to the success of cancer therapy and the improvement of patients’ survival rate.

In the last decade, liquid biopsy has arisen as a promising non-invasive strategy for cancer diagnosis to overcome the limitations of traditional techniques. Liquid biopsy refers to the analysis of tumor biomarkers isolated from biological fluids of cancer patients [2]. The peripheral blood of a cancer patient contains a pool of cells and cell products derived from the tumor, including circulating tumor cells (CTCs), platelets, circulating free (cf) DNA or RNA, exosomes, proteins and metabolites [3,4,5,6]. All this information makes liquid biopsy an interesting high-information-content technique for its application in early detection of cancer, in risk estimation for metastatic relapse, and cancer evolution and treatment response monitoring [7], avoiding invasive methods of sample collection, such as a tissue biopsy.

Mass spectrometry [8,9], calorimetry [10,11,12,13,14], and fluorescence spectroscopy [15] are some of the biophysical techniques that have been used for cancer patient diagnosis by analyzing blood serum. In the case of fluorescence, it is a useful technique traditionally employed for characterizing the structure of biomolecules and its interactions. Detection by fluorescence spectroscopy requires excitation of the biological sample with a radiation beam at a certain wavelength, which promotes fluorescence emission from the different components of the biological sample (mainly macromolecules present in the sample) at larger wavelengths [16]. When there is a pathological state, biochemical, physicochemical, and histological changes occur, and they are reflected in tissue or serum composition. Therefore, it could be reasonable to think that an altered fluorescence emission spectrum would be detected. On this basis, several studies have been published showing that fluorescence spectroscopy can be applied to the diagnosis of tumor diseases and reported that when comparing samples from individuals with and without pathology, the fluorescence properties are different [17,18]. This phenomenon has been studied in both solid tissue [19,20] and serum samples. Breast, cervix, colon, and oropharyngeal cancer [21,22,23,24] were some of the pathologies studied.

Advances in nanotechnology and nanoscience have led to the creation of novel horizons for the use of nanomaterials in cancer diagnosis. Among these, dendrimers (three-dimensional hyperbranched polymers) have gathered increasing attention because of their unique structural properties, including monodispersity, internal molecular cavities, tunable size, and numerous peripheral functional groups [25,26]. The use of dendrimers as nanocarriers for drug and contrast agent delivery for cancer treatment and diagnostic imaging has been intensively investigated by several research groups [27,28,29]. More recently, the multivalency of dendrimers has attracted research interest for the fabrication of dendrimer-based biosensors for biomarker detection. The multiple functional groups on the surface of dendrimers are used to immobilize antibodies or aptamers combined with highly sensitive electrochemical or fluorescence methods for specific tumor biomarker detection [30,31,32,33,34,35,36]. However, the use of dendrimers for biomarker detection purposes can also be figured out through the direct interaction of dendrimers with proteins and biomolecules in a biological fluid, thus approaching the principles of liquid biopsy mentioned above. Indeed, it is well studied that dendrimers, as other nanomaterials, interact with proteins and other biomolecules in a biological fluid, and this provokes the formation of the so-called protein corona around them. Although this phenomenon can bring limitations to drug delivery applications [37], interest has been reported for exploiting it for a variety of medical applications [38] and, particularly, for diagnosis purposes [39,40].

**Figure 1:**
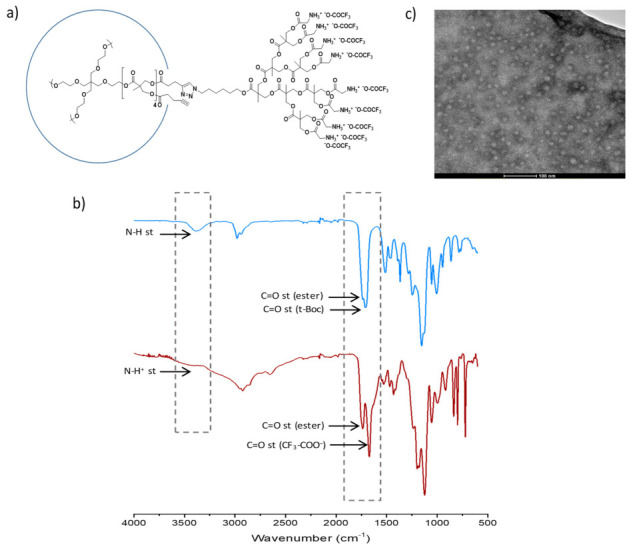
(**a**): Chemical structure of the dendronized hyperbranched polymer DHP-bMPA; (**b**): FTIR spectra of DHP-bMPA compared to its t-Boc-protected precursor. (**c**) TEM image of DHP-bMPA.

The interaction between dendrimers and proteins in blood depends on the peripheral features of the dendrimer, such as positive or negative charge [41], and also the characteristics of protein contents of a given blood sample, and this can be detected by fluorescence spectroscopy [42,43,44]. Typically, tryptophan residues give rise to fluorescent emission upon excitation at 330 nm, and protein modifications around them cause fluorescence changes (either changes in emission intensity or characteristic emission wavelength) that can be detected [45]. This was taken as the basis of this study, in which we assessed a novel and simple approach for cancer detection using a dendronized hyperbranched polymer based on bis(hydroxymethyl)propionic acid (DHP-bMPA) (Figure 1a). DHP-bMPA is based on a fourth generation hyperbranched polymer of 2,2-bis(hydroxymethyl)propionic acid, bis-MPA [46] conjugated at its periphery with third generation bis-MPA dendrons bearing eight terminal glycine moieties. Previous studies conducted by our group demonstrated that some cationic DHP-bMPA derivatives were suitable for DNA transfection [47] and drug delivery [48]. However, their potential as a diagnostic tool remains to be explored [49].

The hypothesis underlying our work is: (1) pathology alters the composition of blood serum (protein content by down- and up-regulation of certain disease-associated proteins, protein modification, and protein-associated metabolites); (2) dendrimers interact differentially with those proteins exhibiting an altered pattern due to the disease; and (3) fluorescence spectroscopy provides discriminating information for these phenomena (fluorescence liquid biopsy, FLB). Here, DHP-bMPA was used in combination with fluorescence spectroscopy in order to improve the classification between cancer and healthy subjects. In this pilot study, 20 pancreatic ductal adenocarcinoma (PDAC) samples and 24 ovary cancer samples were analyzed and compared with their healthy counterparts. Fluorescence spectroscopy was performed in the presence or absence of DHP-bMPA and a biparametric analysis of the fluorescence emission curves was carried out in order to obtain a single fluorescence spectroscopy score (FS_score_) to assess blood serum alterations and predict risk of disease (PDAC or ovarian cancer). The addition of DHP-bMPA resulted in an improved classification of subjects, with an overall improvement in statistical specificity and sensitivity of the classification model in both cancer patient cohorts, when compared with the model elaborated in the absence of dendrimer. Despite the low number of samples considered in this study, this novel strategy exhibited excellent potential for improving clinical cancer diagnostic in a quick, minimally-invasive, and cost-effective manner.

## 2. Results and Discussion

### 2.1. Synthesis and Morphological Characterization of DHP-bMPA

The dendronized hyperbranched polymer, DHP-bMPA, used for this study was synthesized from a tert-butyloxycarbonyl (t-Boc) protected DHP-bMPA precursor bearing an average of 424 t-BocNH groups, which was previously prepared and reported by our group [45]. The amino groups were deprotected with trifluoraoacetic acid. The t-Boc cleavage reaction was followed by Fourier transform infrared (FTIR) spectroscopy (Figure 1b). The chemical structure of the trifluoroacetate product, DHP-bMPA (Figure 1a) was confirmed by nuclear magnetic resonance (NMR) and FTIR spectroscopy (Materials and Methods).

The morphological characterization of DHP-bMPA was carried out by transmission electron microscopy (TEM). The sample was prepared by depositing a 0.5 mg mL^−1^ solution of DHP-bMPA in PBS (phosphate-buffered saline) on a copper grid. DHP-bMPA appeared as rounded objects with average diameter of 15.9 ± 2.2 nm calculated from TEM images (Figure 1c). This size is consistent with the expected formation of unimolecular micelles well dispersed in aqueous solution, and in reasonable agreement with dynamic light scattering determinations (Supplementary Table S1). No aggregation, turbidity, or color changes were observed upon mixing DHP-bMPA with serum.

### 2.2. Preliminary Fluorescence Studies

**Figure 2:**
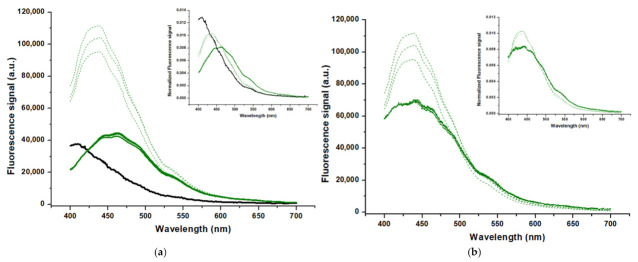
(**a**) Comparison of fluorescence spectra of the DHP-bMPA at 500 µg mL^−1^ concentration (black line) and three blood donors (BD) (green lines) in the absence (solid lines) and in presence (dotted lines) of DHP-bMPA in the medium. (**b**): Comparison of raw fluorescence spectra of three BD samples (green lines) and sum of DHP-bMPA and BD serum fluorescence signal (dotted green lines) of DHP-bMPA in the medium. Fluorescence measurements were performed at excitation wavelength of 330 nm and emission spectra from 400 to 700 nm. Normalized fluorescence signals are represented in the Inset graph.

At 330 nm wavelength, tryptophan residues in proteins are excited and emission fluorescence curves recorded from 400 to 700 nm compile the information of the proteins’ aromatic residues and their environment (neighboring residues exposure to solvent molecules). All serum proteins and their potential interactions with other serum proteins and metabolites would be reflected in this fluorescence spectrum [45]. In order to set up experimental conditions, preliminary fluorescence studies were first carried out with a set of blood donors (BD) samples (three different samples taken as example from the BD group), both in the absence (Figure 2a, solid green lines) and presence of DHP-bMPA (Figure 2a, dotted green lines). The fluorescence spectrum of BD in PBS shows a maximum at 460 nm. In the presence of DHP-bMPA, at a concentration of 500 µg mL^−1^, the contribution to fluorescence was centered around 400 nm (Figure 2a, black line). The fluorescence profile of BD/DHP-bMPA complex was sharped and shifted to lower wavelength (Figure 2a, dot green line). Also, BD/DHP-bMPA profiles differed in terms of intensity at the maximum emission wavelength (Figure 2a dot green lines), in contrast to the BD fluorescence profiles (without DHP-bMPA) that were very similar for all of them (Figure 2a, solid green lines). This could reflect that a different interaction between DHP-bMPA and the protein content of BD samples occurred as they were extracted from different subjects, and this leads to an inherent interindividual variability. Also, the normalization of the fluorescence spectra highlighted a signal shift to lower wavelength values in the case of BD/DHP-bMPA (Inset in Figure 2a).

In an attempt to study further the origin of these differences, the next step consisted in comparing the experimental fluorescence signal of BD/DHP-bMPA and the theoretical fluorescence signal obtained as the sum of both fluorescence spectra, BD and DHP-bMPA, recorded separately. Both types of spectra are plotted in Figure 2b. It can be observed that the experimental spectra recorded for the three BD/DHP-bMPA samples cannot be explained by the sum of the individual fluorescent spectra of DHP-bMPA and BD, and this is consistent with the existence of interaction of DHP-bMPA with the protein content in BD samples. Furthermore, there is a displacement at 525–550 nm between the experimental and theoretical fluorescence maxima (Figure 2b, Inset) that cannot be explained by the DHP-bMPA signal contribution. This shift could be related to BD protein residues specifically contributing to the fluorescence of the sample in that wavelength range and that can be altered upon interacting or binding to DHP-bMPA.

These preliminary results revealed that some serum components interact with DHP-bMPA, resulting in changes in the environment of aromatic residues of serum proteins, and these changes could be detected using fluorescence spectroscopy. Accordingly, we planned to study if this interaction measured by fluorescence might be used for detecting serum protein content differences triggered by the disease and this could lead to distinguish between healthy controls and a certain pathological status based on serum samples analysis.

### 2.3. Fluorescence Studies of Patient Serum Samples

Two different pathologies, pancreatic ductal adenocarcinoma cancer (PDAC) and ovarian cancer (OV), were studied to explore the applicability of the interaction with DHP-bMPA to detect a pathological status based on fluorescence spectra.

#### 2.3.1. Pancreatic Ductal Adenocarcinoma Cancer (PDAC) Serum Samples with and without DHP-bMPA

**Figure 3:**
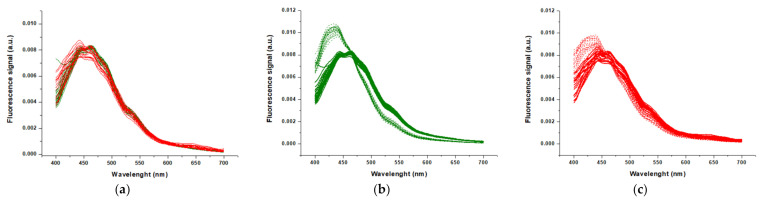
(**a**) Raw fluorescence spectra curves of BD (green lines) and PDAC (red lines); (**b**) Fluorescence spectra of BD samples in the absence (green lines) and presence of DHP-bMPA (dotted green lines); (**c**) PDAC samples in the absence (red lines) and presence of DHP-bMPA (dotted red lines). Serum was diluted 1:25 in PBS, with DHP-bMPA concentration of 500 µg mL^−1^, excitation wavelength 330 nm, and emission spectra from 400 to 700 nm.

Figure 3 gathers the fluorescent spectra of PDAC group in the absence and presence (PDAC/DHP-bMPA) of DHP-bMPA, both compared to BD group. In the absence of DHP-bMPA, no changes in the fluorescence spectra recorded were observed between PDCA and BD (Figure 3a). In contrast, the presence of DHP-bMPA clearly affects the fluorescent behavior of both the BD group, (Figure 3b) and the PDAC serum sample, PDAC/DHP-bMPA (Figure 3c).

**Figure 4:**
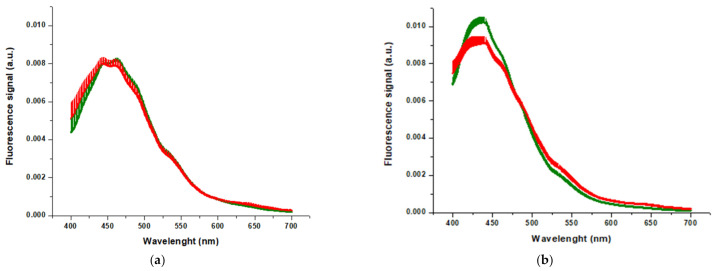
Mean and standard deviation values of fluorescence spectra of serum BD (green) and PDCA samples (red) in absence (**a**) and presence of DHP-bMPA (**b**). Serum was diluted 1:25 in PBS, with DHP-bMPA concentration of 500 µg mL^−1^, excitation wavelength 330 nm, and emission spectra from 400 to 700 nm.

When comparing mean curves signal values (Figure 4a,b), it is clear that the presence of DHP-bMPA promoted changes in the shape (around 525 nm) and the maximum value of fluorescence signals (about 10% increase), which are different for BD and PDAC samples (Figure 4b). This indicates a different interaction was promoted between DHP-bMPA and both BD and PDAC serum samples, indicating the possibility of using this strategy with diagnostic purposes.

#### 2.3.2. Ovarian Cancer (OV) Serum Samples with and without DHP-bMPA

**Figure 5:**
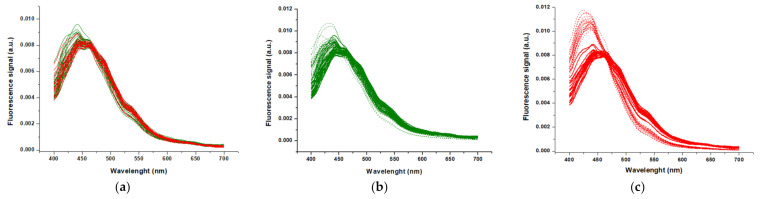
(**a**) Raw fluorescence spectra curves of OC (green lines) and OV (red lines); (**b**) Fluorescence spectra of OC samples in the absence (green lines) and presence of DHP-bMPA (dotted green lines); (**c**) OV samples in the absence (red lines) and presence of DHP-bMPA (dotted red lines). Serum was diluted 1:25 in PBS, with DHP-bMPA concentration of 500 µg mL^−1^, excitation wavelength 330 nm, and emission spectra from 400 to 700 nm.

Figure 5 gathers the fluorescence spectra of ovarian cancer (OV), serum samples in the absence (Figure 5a) and presence of DHP-bMPA (Figure 5b), both compared to their respective benign ovarian cyst women as controls (OC). As happened for PDAC samples, no changes in the fluorescence spectra recorded were observed between the disease state and OC in the absence of DHP-bMPA (Figure 5a). In contrast, the presence of DHP-bMPA affects the fluorescent behavior of both OC (Figure 5b) and OV (Figure 5c) samples in a different way.

**Figure 6:**
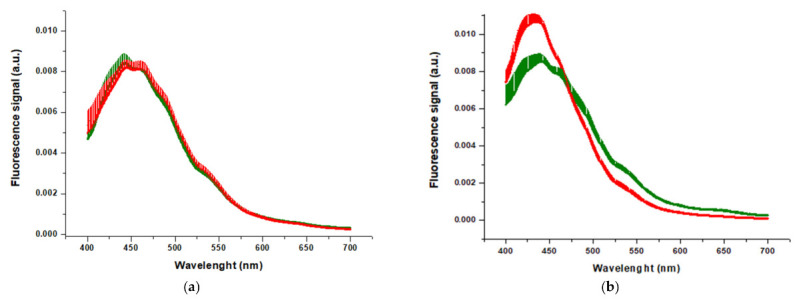
Mean and standard deviation values of fluorescence spectra of serum OC (Green) and OV samples (Red) in absence (**a**) and presence of DHP-bMPA (**b**). Serum was diluted 1:25 in PBS, with DHP-bMPA concentration of 500 µg mL^−1^, excitation wavelength 330 nm, and emission spectra from 400 to 700 nm.

When comparing mean curves signal values (Figure 6a,b), it is apparent that the presence of DHP-bMPA promoted changes in the shape (around 525 nm) and the maximum value of fluorescence signals (about 20% increase) only in the OV group (Figure 6b). OC samples spectra did not change with DHP-bMPA incubation. Again, as it occurred in PDAC disease samples, DHP-bMPA promoted different changes in OC and OV, thus supporting the objective of using fluorescence spectroscopy of serum samples to assess disease-induced blood serum alterations and assign a probability of cancer from serum samples.

### 2.4. Evaluation of the Potential Diagnostic Capability of DHP-bMPA

According to the results described above, DHP-bMPA seems to interact with proteins from serum samples producing different responses (changes in fluorescence emission) in PDAC and OV samples compared to their respective BD and OC control groups. These observations fostered the statistical evaluation of fluorescence spectra in order to confirm the possibility of using the incubation treatment with DHP-bMPA to discriminate between healthy and disease status. This would impart DHP-bMPA with diagnostic capability.

#### 2.4.1. Fluorescence Curves Parametrization and Statistical Analysis Applied to PDAC Samples

##### Fluorescence Signals Parametrization

The fluorescence signal was analyzed by calculating two parameters: *G*_1_ and *WL*_max_ (according to Equations (1) and (2) in Section 3). The skewness, *G*_1_, describes the asymmetry of the spectrum, and *WL*_max_ is the wavelength for the maximal intensity in the spectrum. The methodology we developed was protein concentration-independent (avoiding the problems derived from protein concentration determinations). A complete descriptive analysis of the parameters can be found in Supplementary Table S2.

##### Statistical Analysis of Parameters: Univariate Analysis

Thus, DHP-bMPA promoted changes in *G*_1_ values of BD, with an increase of 35%, and PDAC, with an increase of 20%. *WL*_max_ values decreased 23 nm in BD and 5.5 nm in PDAC, and this change seemed to be the reason for the loss of significance between BD and PDAC previously observed. Based on these data, the effect of DHP-bMPA in presence of serum samples could be more remarkable in BD group.

**Figure 7:**
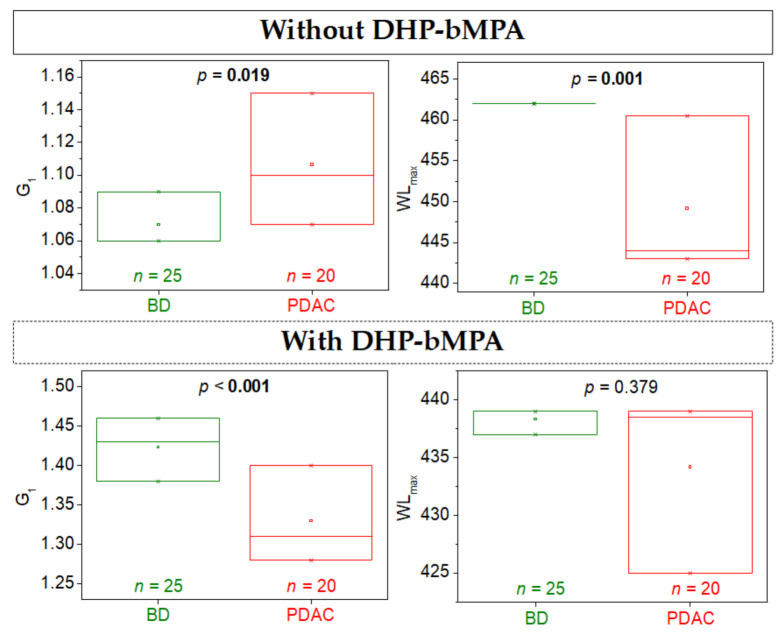
Boxplots of fluorescence spectra-associated parameters (*G*_1_ and *WL*_max_) in each clinical group (BD and PDAC), in the absence or presence of DHP-bMPA. The *p*-values were calculated according to Wilcoxon test.

The statistical analysis of *G*_1_ and *WL*_max_ parameters from fluorescence spectra of BD and PDAC serum samples was performed. Despite the apparent similarity of the fluorescence spectra of the BD group and PDAC patients in the absence of DHP-bMPA (Figure 3a and Figure 4a), there were statistically significant differences (*p* < 0.050) in fluorescence curves-associated parameters (*G*_1_ and *WL*_max_) when comparing both BD and PDAC (Supplementary Table S3). When DHP-bMPA was added to serum samples (Figure 3b,c and Figure 4a), fluorescence curves-associated parameters changed (Supplementary Table S3): *G*_1_ maintained its significant difference between groups (*p* < 0.050), but *WL*_max_ increased its *p*-value to 0.379 (losing statistical significance). If we have a deep look to the parameter values, *WL*_max_ mean values without DHP-bMPA were 18 nm higher in BD, and *G*_1_ was 0.04 lower in PDAC patients. Accordingly, the presence of DHP-bMPA enhanced the difference in the mean values of *G*_1_ (∆*G*_1_ = 0.12) and brought closer *WL*_max_ values between BD and PDAC, until they were almost the same (∆*WL*_max_ = 1.50 nm) (Figure 7).

Thus, DHP-bMPA promoted changes in *G*_1_ values for BD, with an increase by 35%, and PDAC, with an increase by 20%. *WL*_max_ values decreased by 23 nm in BD and by 5.5 nm in PDAC, and this change seemed to be the reason for the loss of statistical significance between BD and PDAC previously observed. Based on these data, the effect of DHP-bMPA on serum samples could be more remarkable in the BD group.

##### Statistical Analysis of Parameters: Multivariate Analysis

As described above, monovariant analysis of the individual parameters (*G*_1_ and *WL*_max_) obtained from the fluorescence spectra showed that they were statistically different (*p*-value < 0.050). We constructed a classification model with these two parameters and calculated a single classification index, called FS_score_ (Fluorescence Spectrum Score), for classifying samples from each group (BD vs. PDAC), following a methodology previously described in our group [12].

We elaborated two scores calculated on the basis of the parameters *G*_1_ and *WL*_max_, FS_score_ and FS_score_-DHP-bMPA, (that is, FS_score_ for samples untreated and treated with DHP-bMPA, respectively) and evaluated their capability to classify subjects and, therefore, predict risk of PDAC. The results, gathered in Table 1, indicated that *G*_1_ and *WL*_max_ parameters included in FS_score_-DHP-bMPA were statistically significantly different (*p* < 0.050), while in FS_score_ only the *WL*_max_ parameter was statistically significant. Therefore, when using DHP-bMPA, the model was improved.

The values of the FS_score_ comprises a range between 0 and 1. Typically, a 0.5 threshold is used. FS_score_ values > 0.5 indicate that the model is classifying a serum sample as altered (that is, reflecting PDAC disease), whereas FS_score_ values < 0.5 indicate that the model was classifying a serum sample as non-altered (that is, not reflecting any disease, closer to the BD group).

A good performance of the model corresponds to maximizing the number of true positives and negatives and minimizing the number of false positives and negatives. The results indicated that when using DHP-bMPA, the model performed considerably better. That is, there were less false positives and negatives. The number of true negatives (FS_score_-DHP-bMPA < 0.5 in BD group) and true positives (FS_score_-DHP-bMPA > 0.5 in PDAC patients) increased in comparison to the FS_score_-based model. Similarly, the number of false positives (FS_score_-DHP-bMPA > 0.5 in BD group) and false negatives (FS_score_-DHP-bMPA < 0.5 in PDAC patients) decreased in comparison to the FS_score_-based model (Table 2).

**Figure 8:**
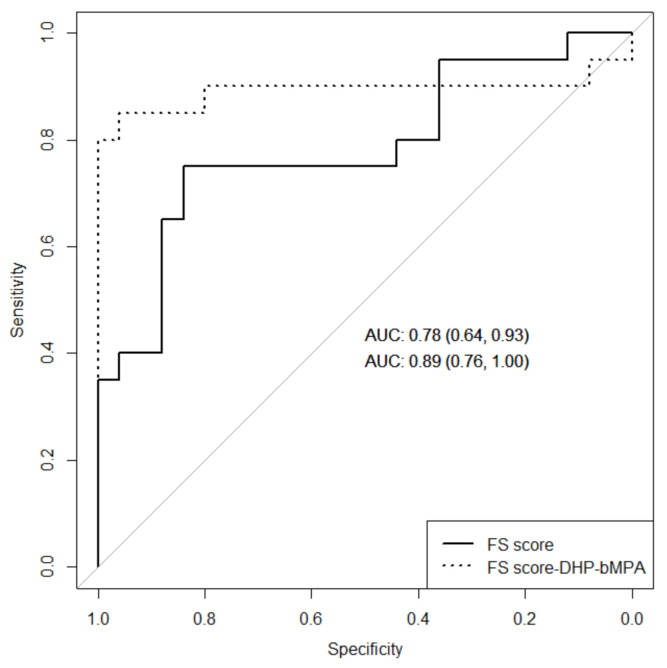
ROC curve illustrating the statistical performance of FS_score_ and FS_score_-DHP-bMPA for predicting risk of PDAC.

Summarizing the statistical performance, FS_score_ exhibited good performance indexes, according to the success rate (75.6%) and area under the Receiver Operating Characteristic (ROC) curve (0.78 AUC), with better specificity (88.0%) than sensitivity (60.0%) (Figure 8, ROC curve, Table 3). When using DHP-bMPA, FS_score_-DHP-bMPA improved all indexes, with better success rate (91.1%), larger AUC (0.89), and higher specificity (96.0%) and sensitivity (85.0%). In addition, according to the Net Reclassification Index (NRI) of Pencina [50], 33.0% of patients were more correctly reclassified using FS_score_-DHP-bMPA compared to FS_score_. This reclassification involved mainly the PDAC group (25.0%), not the BD group (8.0%).

**Figure 9:**
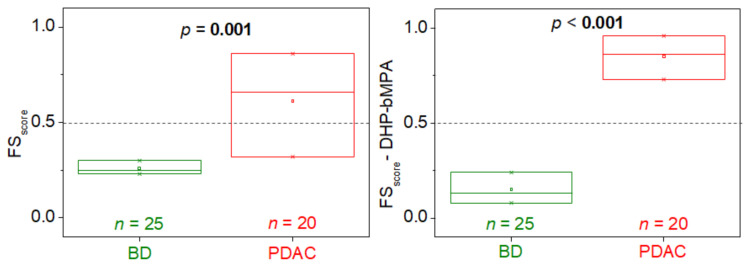
Distribution of the probability score (FS_score_ and FS_score_-DHP-bMPA) within BD (**green**) and PDAC (**red**) groups. The *p*-value (Wilcoxon test) indicates there is a statistically significant difference between BD group and PDAC patients (*p*-value < 0.050).

The statistical analysis of the FS_score_ values showed significant differences between BD and PDAC group (*p* = 0.001). Differences were higher for FS_score_-DHP-bMPA (*p* < 0.001) (Supplementary Table S4, Figure 9).

**Figure 10:**
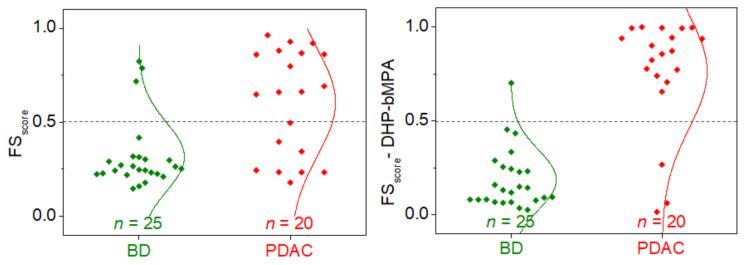
Distribution of the FS_score_ (**left**) and FS_score_-DHP-bMPA (**right**) within BD (**green**) and PDAC (**red**) groups. The lines represent an equivalent Gaussian distribution. The FSscore threshold for discriminating between an unaltered and altered fluorescence spectrum is 0.5 (dotted horizontal line).

The improvement in the predictive capability of FS_score_ when DHP-bMPA was employed can be observed in Figure 10. There were three BD subjects with FS_score_ > 0.5 (12.0% false positive rate) and eight PDAC patients with FS_score_ < 0.5 (40.0% false negative rate). Data were improved by using DHP-bMPA: 1 BD subject with FS_score_-DHP-bMPA > 0.5 (4.0% false positive rate) and three PDAC patients with FS_score_-DHP-bMPA < 0.5 (6.7% false negative rate).

#### 2.4.2. Fluorescence Curves Parametrization and Statistical Analysis Applied to OV Samples

##### Fluorescence Signals Parametrization

As in PDAC disease, the fluorescence spectra were analyzed by calculating the same two parameters, *G*_1_ and *WL*_max_. A complete descriptive analysis of the parameters can be found in Supplementary Table S5.

##### Statistical Analysis of Parameters: Univariate Analysis

**Figure 11:**
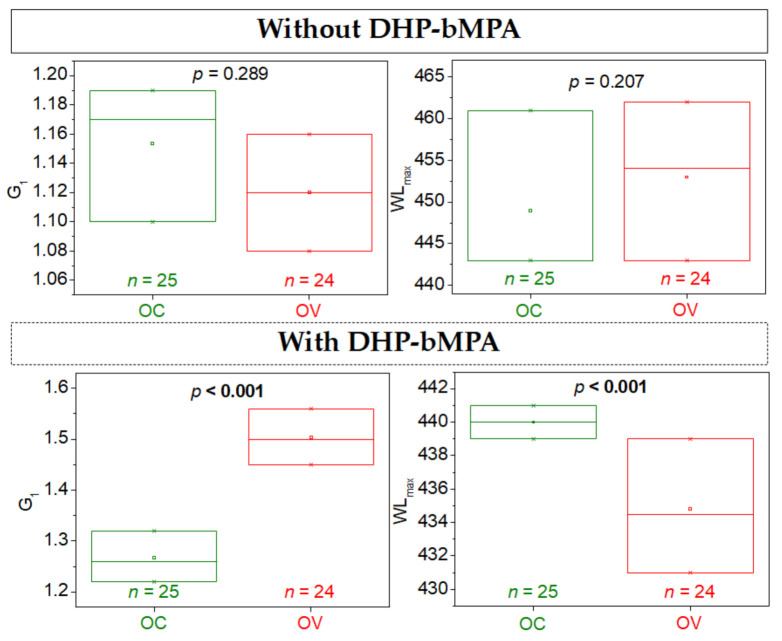
Boxplots of fluorescence spectra-associated parameters in each clinical group, OC and OV, in the absence or the presence of DHP-bMPA. The *p*-values were calculated according to t-test or Wilcoxon test, depending on the normality character of the parameter distribution.

The statistical analysis of *G*_1_ and *WL*_max_ from fluorescence spectra of OC and OV samples was performed (Supplementary Table S6) and they did not exhibit significant differences (*p* > 0.050). However, the presence of DHP-bMPA promoted changes in G_1_ and *WL*_max_, rendering them statistically different between the two groups, OC and OV (*p* < 0.001) for both parameters. If we analyze the parameters, *WL*_max_ values increased by 3 nm in OC and by 20.5 nm in OV group when DHP-bMPA was employed. The same effect was observed in the case of *G*_1_, which increased by 0.1 in OC and by 0.4 in cancer patients when DHP-bMPA was used. The boxplots (Figure 11) of *G*_1_ and *WL*_max_ comparing both groups, OC and OV samples, reflected the effect of the presence of DHP-bMPA. Based on these results, it could be said that the effect of DHP-bMPA on the fluorescence spectrum of serum samples was more remarkable in OV than in the OC group. Having in mind the previous results regarding PDAC, for which BD spectra were more affected, DHP-bMPA seemed to interact in a different way with serum proteins depending on the disease. This is consistent with a different serum protein composition in different diseased and control subjects.

##### Statistical Analysis of Parameters: Multivariate Analysis

As in the case of PDAC, we constructed two scores calculated on the basis of the two parameters, *G*_1_ and *WL*_max_, FS_score_ and FS_score_-DHP-bMPA, (that is, FS_score_ for samples untreated and treated with DHP-bMPA, respectively) and evaluated their capability to predict risk of ovarian cancer. The results, gathered in Table 4, indicated that *G*_1_ parameter included in FS_score_-DHP-bMPA was statistically significantly different (*p* < 0.050). Therefore, when using DHP-bMPA, the model was improved.

Again, a 0.5 threshold was set (FS_score_ > 0.5 classified a sample as diseased status, and FS_score_ < 0.5 classified a sample as non-diseased status) and the results indicated that the model performed better when using DHP-bMPA. There were more true positives and negatives and less false positives and negatives when using the dendrimer in the experimental protocol; the number of true negatives (FS_score_-DHP-bMPA < 0.5 in OC group) and true positives (FS_score_-DHP-bMPA > 0.5 in OV group) increased compared to FS_score_ model. Similarly, the number of false positives (FS_score_-DHP-bMPA > 0.5 in OC group) and false negatives (FS_score_-DHP-bMPA < 0.5 in OV group) decreased compared to FS_score_ model (Table 5).

**Figure 12:**
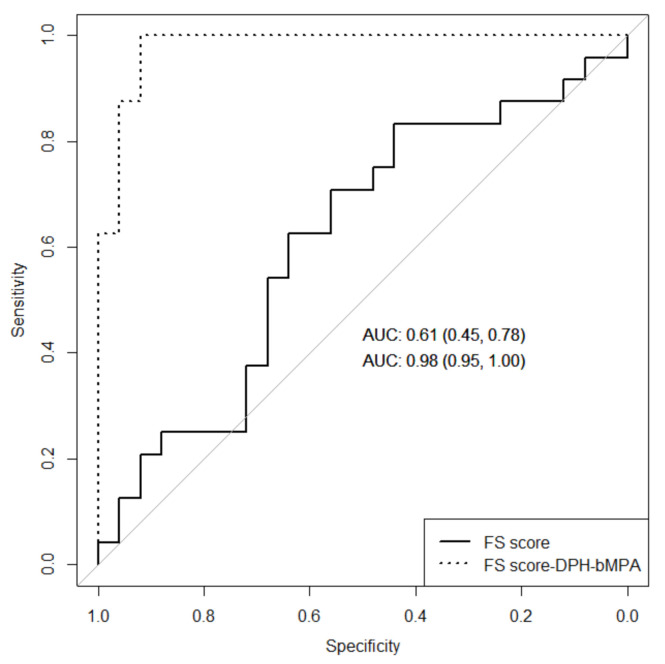
ROC curve illustrating the statistical performance of FS_score_ and FS_score_-DHP-bMPA for predicting risk of ovarian cancer.

FS_score_ showed poor performance indexes, according to success rate (57.1%), area under curve (0.61 AUC), and statistical specificity and sensitivity around 45.8% and 68.0%, respectively (Figure 12, Table 6). When using DHP-bMPA, FS_score_-DHP-bMPA showed much better performance indexes: success rate (91.8%), area under curve (0.98), specificity (92.0%), and sensitivity (91.7%). Also, according to Net Reclassification Index (NRI) of Pencina [50], approximately 70.0% of patients were correctly reclassified using FS_score_-DHP-bMPA. This reclassification involved mainly the OV group (45.8%), and to a lesser extent the OC group (24.0%). Delog test was used to compare areas under curves (AUC), and they were statistically significantly different (*p* < 0.001) in their AUC.

**Figure 13:**
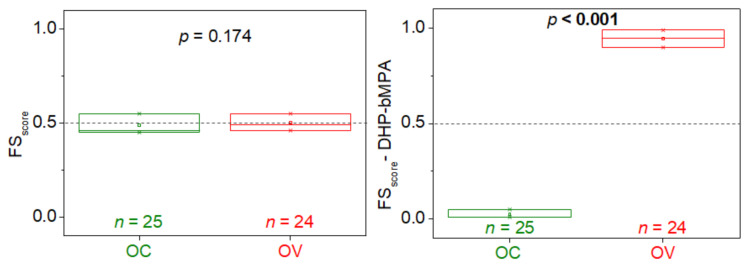
Distribution of the probability score (FS_score_ and FS_score_-DHP-bMPA) within OC (**green**) and OV (**red**) groups. The *p*-value (Wilcoxon test) indicates there is a statistically significant difference between OC and OV groups (*p*-value < 0.001) in FS_score_-DHP-bMPA.

The statistical analysis of the FS_score_ values indicated there were no significant differences between OC and OV groups (*p* = 0.174), but remarkably significant differences for FS_score_-DHP-bMPA (*p*-value < 0.001) (Supplementary Table S7, Figure 13).

**Figure 14:**
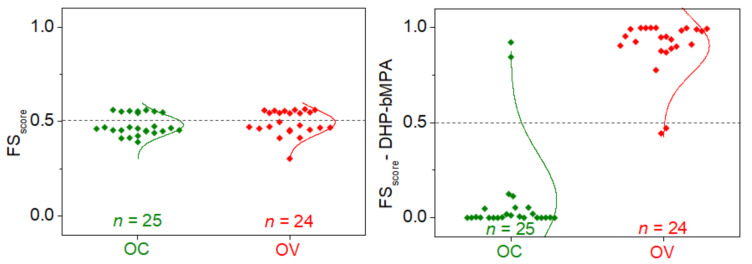
Distribution of the FS_score_ (**left**) and the FS_score_- DHP-bMPA (**right**) within OC (**green**) and OV (**red**) groups. Notes: The lines represent an equivalent Gaussian distribution. The FS_score_ threshold for discriminating between an unaltered and altered fluorescence spectrum is 0.5 (dotted horizontal line).

The excellent improvement in the predictive value of FS_score_ when DHP-bMPA was included in the experimental protocol can be observed in Figure 14. There were 8 OC samples with FS_score_ > 0.5 (32.0% false positive rate) and 13 OV samples with FS_score_ < 0.5 (54.2% false negative rate). The classification was improved by using DHP-bMPA: There were only two OC samples with FS_score_-DHP-bMPA > 0.5 (8.0% false positive rate) and two OV samples with FS_score_-DHP-bMPA < 0.5 (8.3% false negative rate).

## 3. Materials and Methods

### 3.1. Reagents

Unless otherwise indicated, all reagents were purchased from Sigma-Aldrich (Madrid, Spain) and used without further purification. The solvents were purchased from Scharlab, S.L. (Sentmenat, Spain). Tris(benzyltriazolylmethyl)amine (TBTA) catalyst, 4-(dimethylamino) pyridinium 4-toluenesulfonate (DPTS) salt and were prepared in our laboratory.

### 3.2. Synthesis and Characterization of the Dendronized Hyperbranched Polymer DHP-bMPA

**Scheme 1:**
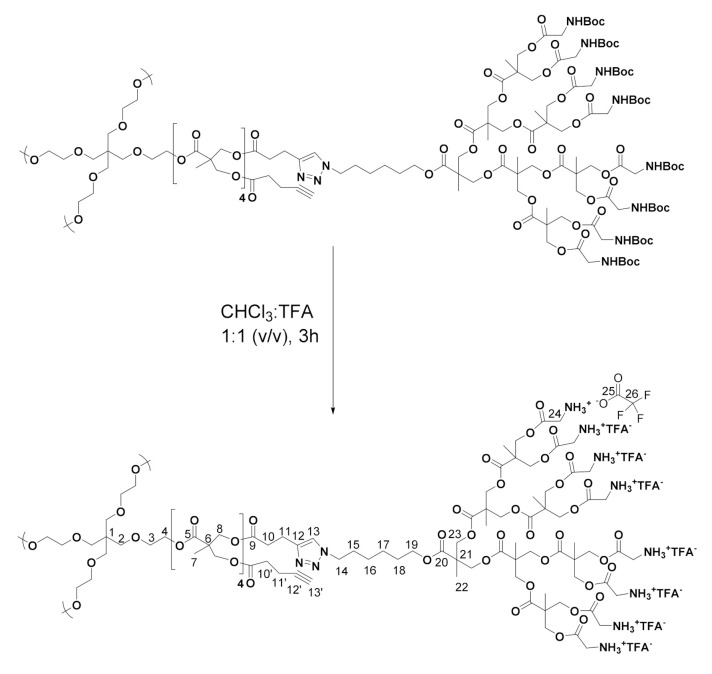
Deprotection step of t-Boc protected DHP-bMPA precursor synthesized as described in ref. [47].

The *t*-Boc-protected dendronized hyperbranched polymer (DHP-bMPA) was synthesized as previously reported by us [47]. Terminal amines were deprotected as depicted in Scheme 1, by dissolving *t*-Boc protected DHP-bMPA (1.00 eq.) in a mixture of chloroform and trifluoroacetic acid (5 mL, 1:1 volume/v). The reaction mixture was stirred at room temperature for 3 h. The solvent and the excess of trifluoroacetic acid were removed under vacuum. Then, the product was dissolved in methanol and precipitated in cold ether. After decanting, the remaining solvent traces were removed under reduced pressure and the product was obtained as a white solid.

^1^H NMR (400 MHz, CD_3_OD) *δ* (ppm): 1.32 (1293H, m, H-7 and H-22), 1.42 (212H, m, H-16 and H-17), 1.70 (106H, m, H-18), 1.92 (106H, m, H-15), 2.73 (106H, m, H-10) 3.00 (106H, m, H-11), 3.67 (16H, m, H-2 and H-3), 3.95 (848H, m, H-24), 4.16 (106H, m, H-19), 4.32 (884H, m, H-4, H-8 and H-23[1,2G]), 4.44 (954H, H-14 and H-23[3G]), 7.80 (53H, s, H-13).

^13^C NMR (100 MHz, C_2_D_6_SO) *δ* (ppm): 16.9, 17.1 (C-7 and C-22), 20.5 (C-11), 24.8 (C-17), 25.7 (C-16), 28.0 (C-18), 29.8 (C-15), 32.8 (C-10), 46.0 (C-24), 46.3 (C-6, and C-21), 49.3 (C-14), 52.7, 63.7, 65.7 (C-8, C-19, C-23), 117.0 (J_C-F_ = 300 Hz, C-26), 122.0 (C-12), 145.2 (C-13), 158.7 (J_C-C-F_ = 31.5 Hz, C-25), 167.4 (C-20[G3]), 167.6, 168.2, 169.1, 171.5, 172.8 (C-5, C-9 and C-20[G0,1,2]).

FTIR (νmax/cm^−1^, ATR): 3450 (bs, N-H^+^ st), 2930, 2860 (C-H st), 1737 (C = O st ester), 1670 (C = O st CF_3_-COO^−^), 1520 (N-H^+^ δ), 1432 (CH2, CH3 *δ*), 1193 (CO-O st), 1122 (O-C-C st).

### 3.3. Dendrimer Characterization Techniques

^1^H and ^13^C Nuclear Magnetic Resonance (NMR) experiments were performed using an AV-400 (^1^H: 400 MHz, ^13^C: 100 MHz) spectrometer (Bruker, Billerica, MA, USA) employing deuterated methanol (CD_3_OD) or deuterated dimethyl sulfoxide ((CD_3_)_2_SO) as solvents. The chemical shifts are given relative to tetramethylsilane (TMS) in ppm and the coupling constants in Hz; as internal standard, the solvent residual peak was used for spectrum calibration. Infrared spectra were recorded between 4000 and 600 cm^–1^ on a Vertex 70 spectrophotometer (Bruker, Billerica, MA, USA), which worked in attenuated total reflection (ATR) mode.

Transmission electron microscopy (TEM) images were recorded on a TECNAI T20 system (FEITM, Hillsboro, OR, USA) with a beam power of 200 kV. A droplet of a solution of the sample at 0.5 mg mL^−1^ in PBS was deposited on a holey carbon film 300 mesh coppered grids (Electron Microscopy Sciences, Hatfield, PA, USA) and a 3% *w/v* aqueous solution of phosphotungstic acid was used as negative stain.

### 3.4. Subjects and Samples

Experimental blood samples were obtained from four different sets, grouped two by two with the appropriate controls (diseased vs. controls): pancreatic cancer patients (PDAC) vs. blood donors (BD); ovarian cancer patients (OV) vs. benign ovarian cyst patients (OC). All subjects gave their informed consent for inclusion before they participated in the study. The study was conducted in accordance with the Declaration of Helsinki, and the protocol was approved by the Ethics Committee of CEICA (PI16/0228).

#### 3.4.1. Pancreatic Cancer (PDAC): Patients (PDAC Diseased Group) and Blood Donor Subjects (BD Control Group) Cohort Sample Description

Samples and data from blood donor subjects as control group patients included in this study were obtained from blood donors at Blood and Tissue Bank of Aragon and provided by the Biobank of the Aragon Health System, integrated in the Spanish National Biobanks Network (PT20/00112). They were processed following standard operating procedures with the appropriate approval of the Ethics and Scientific Committees. The cohort consisted of 25 serum samples from Spanish Caucasian subjects, apparently diseased-free.

A total of 20 serum samples from patients diagnosed with pancreatic cancer at the oncology service at Hospital Universitario Miguel Servet in Zaragoza (Spain) were used in this study. Samples were collected after histopathological confirmation and prior to treatment. They were handled, stored, and provided by Biobank of the Aragon Health System.

Regarding disease/health, 55.6% of the samples were blood donor controls (BD) and 44.4% were patients diagnosed of pancreatic cancer (PDAC). According to the gender, 27/45 (60.0%) were males and 17/45 (37.8%) were females (there was only one missing demographic data in BD group). In the BD group, 16/25 (64.0%) were males and 8/25 (32.0%) were females, while in the PDAC group, 11/20 (55.0%) were males and 9/20 (45.0%) were females. 

The age from PDAC group is statiscally higher than the age from BD group (*p* < 0.001, Wilcoxon test, Table 7). In the BD group, the age from males was statistically higher than the age from females (*p* = 0.036, *t*-test, Table 8). In the PDAC group, there were no statistically significant differences in age depending on gender (*p* = 0.703, Wilcoxon test, Table 8).

#### 3.4.2. Ovarian Cancer (OV): Ovarian Cancer Patients (OV Diseased Group) and Benign Cyst Patients (OC Control Group) Cohort Sample Description

Samples from ovarian cancer (24) and benign cysts (25) patients diagnosed at the gynecology service at Hospital Virgen del Camino in Pamplona (Spain) were included in this study. Serum samples were collected before surgery, handled, stored, and provided by the Biobank Navarrabiomed integrated in the Spanish National Biobanks Network (PT17/0015/0007). They were processed following standard operating procedures with the appropriate approval of the Ethics and Scientific Committees.

We recruited 49 samples, 24/49 (49.0%) were from female patients diagnosed of ovarian cancer (OV), and 25/49 (51.0%) were from female patients with initially suspected ovarian cancer, but finally diagnosed as benign (OC). There was only one missing age data in the OV group.

The age from OC group is statistically higher than age from OC group (*p* = 0.011, Wilcoxon test, Table 9).

### 3.5. Blood Sample Processing

Peripheral blood (10 mL) from each subject was collected and transferred into serum separator tubes for subsequent analysis. Tubes were gently mixed immediately after blood collection and then centrifuged at 3200× *g* rpm for 10 min. Separated serum was carefully aspirated to avoid hemolysis and contamination of the separated blood phases, and immediately stored in 1 mL aliquots at −80 °C until analysis.

### 3.6. Fluorescence Spectroscopy

Serum samples were diluted 1:25 in PBS and, when appropriate, mixed with DHP-bMPA nanoparticles to a final concentration of 500 µg mL^−1^ prior data acquisition. A total volume of 80 µL was transferred into 96-well microplates (96-well PCR plate, non-skirted, from 4titude, Surrey, UK). Fluorescence measurements were performed in a CLARIOstar plate reader (BMG Labtech, Germany) selecting an excitation wavelength of 330 nm, with excitation and emission bandwidths of 10 nm, and recording the fluorescence emission spectra in the range 400–700 nm. The excitation wavelength was slightly higher than optimal for tryptophan excitation (295 nm), but was a constraint from the instrument. Multimode plate readers with fluorescence capabilities very seldom offer the possibility to use lower excitation wavelengths.

All the fluorescence spectra, *F*(*λ*), where *F* is the fluorescence emission intensity and *λ* is the wavelength, were normalized to an area equal to 1, and two parameters (*G*_1_ and *WL*_max_) were calculated according to the following equations:

(1) $$\begin{array}{c} m_{k}=\frac{\sum_{j}F\left(\lambda_{j}\right){\left(\lambda_{j}-\lambda \right)}^{k}}{\sum_{j}F\left(\lambda_{j}\right)}\\ G_{1}=\frac{m_{3}}{m_{2}^{3/2}} \end{array}$$

where <*λ*> is the average wavelength (or first moment) considering the spectrum as a probability density function:

(2) $$\lambda =\frac{\sum_{j}F\left(\lambda_{j}\right)\lambda_{j}}{\sum_{j}F\left(\lambda_{j}\right)}$$

*m*_2_ and *m*_3_ are the second and third moments of that density distribution, *G*_1_ is the parameter that provides a measure of the asymmetry or skewness of the spectrum. In addition, the wavelength corresponding to the maximal fluorescence intensity of the spectrum (mode of the probability density function), *WL*_max_, is a central measure providing information about the location of the main region of the spectrum.

### 3.7. Statistical Analysis

Fluorescence raw data were processed using Origin software (OriginLab, Northampton, MA). Two parameters summarizing the essential geometric features of each fluorescence spectrum were directly calculated, *G*_1_ and *WL*_max_, and were used for calculating the FS_score_ to compare fluorescence spectra and build the classification model by using a generalized linear model (GLM). FS_score_ provides the probability of an individual fluorescence spectrum for reflecting serum alterations. Such FS_score_ is a single value employed for classifying a given subject as control or diseased. As any probability, the FS_score_ ranges between 0 and 1: the model classifies the subjects with FS_score_ values > 0.5 as individuals with high probability of having serum alterations (i.e., diseased status), and the subjects with FS_score_ values < 0.5 as individuals with low probability of having serum alterations (i.e., healthy status). The performance of the diagnostic test was evaluated by calculating common statistical performance indexes (e.g., sensitivity, specificity) and the receiver operating characteristic (ROC) curve. Net Reclassification Index (NRI) of Pencina and/or Delong test were performed to compare area under curve (AUC) ROC.

The Kolmogorov–Smirnov test was performed to assess the normal distribution of the variables. Averages between two independent groups were compared with the t-test in normal distributions. Medians between two independent groups were compared with the Wilcoxon test in non-normal distributions. Comparisons between groups for qualitative variables were made using a chi-squared test.

The statistical analyses were performed using Rstudio, R version 3.6.1 (RStudio, PBC, Boston, MA, USA) (2019-07-05). For all tests, a two-sided *p*-value of less than 0.050 was considered statistically significant.

## 4. Conclusions

There is an urgent necessity for an efficient, reliable, quick, non-invasive diagnostic test for cancer patient management, applicable not only for early diagnosis, but also for patient stratification and prioritization, as well as personalized patient surveillance and monitoring. New diagnostic methods can be created by applying newly developed experimental techniques or by improving traditional ones through new technological or analytical developments. In this work, we present a novel diagnostic test consisting of using the fluorescence emission signal obtained from serum samples of patients with cancer (PDAC or ovary) by adding a specific type of dendritic polymer (a cationic dendronized hyperbranched polymer, DHP-bMPA). The differences observed in the fluorescence emission of patients’ serum samples compared to the controls were parametrized in two spectra features: the skewness (*G*_1_) and the wavelength at the maximum signal value (*WL*_max_). The statistical analysis of these parameters led us to define two scores, FS_score_ and FS_score_-DHP-bMPA (without and with DHP-bMPA, respectively). Accordingly, we were able to increase the performance of the test for classifying individuals into healthy and cancer groups with very good indicators (accuracy, specificity and sensitivity). It must be emphasized that this is a quick, minimally invasive, risk-free test that can be applied routinely, and it only requires a sample obtained from a blood draw and a fluorescence plate reader, a non-expensive equipment available at many clinical laboratories. The findings herein reported set the basis for an easy and versatile approach to liquid biopsy that combines fluorescence spectroscopy and nanomaterials. These are preliminary results that open a larger study to move fluorescence spectroscopy closer to the clinical application, providing useful information to the oncologist during patient diagnosis.

## Figures and Tables

**Table 1:** Summary of the application of Binomial Generalized Linear Model with Logistic Regression (GLM) to the two FS_score_ for predicting risk of PDAC.

Model	DHP-bMPA	Parameter	*z*-Value	*p*-Value
FS_score_	without	*G*_1_	1.07	0.285
		*WL*_max_	−2.02	**0.043**
FS_score_- DHP-bMPA	with	*G*_1_	−3.51	**<0.001**
		*WL*_max_	−2.41	**0.016**

A cut-off value of 2 for *z*-value (which approximately corresponds to a two-sided hypothesis test with a significance level of *α* = 0.05) indicates whether the corresponding parameters are statistically meaningful in the model. In other words, if the absolute value of the *z*-value is larger than 2, then its corresponding GLM coefficient is non-negligible, i.e., not null, and the parameter is important and meaningful for the prediction of the presence/absence of PDAC cancer.

**Table 2:** Contingency table for predicting risk of PDAC in a BD group.

Reference	Predicted	
	FS_score_	
	<0.5	>0.5
BD (*n* = 25)	22	3
PDAC (*n* = 20)	8	12
	**FS_score_-DHP-bMPA**	
	**<0.5**	**>0.5**
BD (*n* = 25)	24 (↑)	1 (↓)
PDAC (*n* = 20)	3 (↓)	17 (↑)

↑ means that the value increases when using DHP-bMPA; ↓ means that the value decreases when using DHP-bMPA.

**Table 3:** Model comparison based on the ability to predict risk of PDAC.

Indexes	FS_score_	FS_score_-DHP-bMPA
Success Rate	75.56%	91.11%
Sensitivity	60.00%	85.00%
Specificity	88.00%	96.00%
AUC (95% CI)	0.78 (0.64, 0.93)	0.89 (0.76, 1.00)
NRI (95% CI)	n.a.	33.00% (0.02, 0.64)

AUC: area under curve; NRI: net reclassification index; CI: confidence interval; n.a.: not applicable.

**Table 4:** Summary of the application of Binomial Generalized Linear Model with Logistic Regression (GLM) to the two FS_score_ for predicting risk of ovarian cancer.

Model	DHP-bMPA	Parameter	*z*-Value	*p*-Value
FS_score_	without	*G*_1_	−0.19	0.851
		*WL*_max_	0.46	0.646
FS_score_-DHP-bMPA	with	*G*_1_	2.78	**0.005**
		*WL*_max_	1.76	0.078

A cut-off value of 2 for *z*-value (which approximately corresponds to a two-sided hypothesis test with a significance level of *α* = 0.05) indicates whether the corresponding parameters are statistically meaningful in the model. In other words, if the absolute value of the *z*-value is larger than 2, then its corresponding GLM coefficient is non-negligible, i.e., not null, and the parameter is important and meaningful for the prediction of the presence/absence of ovarian cancer.

**Table 5:** Contingency table for predicting risk of OV in the OC group.

Reference	Predicted	
	FS_score_	
	<0.5	>0.5
OC (*n* = 25)	17	8
OV (*n* = 24)	13	11
	**FS_score_-DHP-bMPA**	
	**<0.5**	**>0.5**
OC (*n* = 25)	23 (↑)	2 (↓)
OV (*n* = 24)	2 (↓)	22 (↑)

Note: **↑**means that the value increases when using DHP-bMPA; **↓** means that the value decreases when using DHP-bMPA.

**Table 6:** Model comparison based on the ability to predict risk of ovarian cancer.

Indexes	FS_score_	FS_score_-DHP-bMPA
Success Rate	57.14%	91.84%
Sensitivity	45.83%	91.67%
Specificity	68.00%	92.00%
AUC (95% CI)	0.61 (0.45, 0.78)	0.98 (0.95, 1.00)
p-value Delong test for AUC	n.a.	<0.001
NRI (95% CI)	n.a.	69.80% (0.36, 1.03)

AUC: area under curve; NRI: net reclassification index; CI: confidence interval; n.a.: not applicable.

**Table 7:** Descriptive of age depending on clinical group, blood donor (BD), and pancreatic cancer (PDCA).

Clinical Group	Minimum	Q1	Q2	Average	Q3	Maximum
BD (*n* = 24)	30.00	39.50	49.50	47.92	56.25	68.00
PDAC (*n* = 20)	49.00	60.00	68.00	65.05	70.25	75.00
Overall (*n* = 44)	30.00	48.50	55.00	55.70	68.00	75.00

**Table 8:** Descriptive of age depending on gender in each clinical group (BD and PDCA).

**BD**	**Minimum**	**Q1**	**Q2**	**Average**	**Q3**	**Maximum**
Males (*n* = 16)	33.00	46.25	51.00	51.62	57.25	68.00
Females (*n* = 8)	30.00	30.00	39.00	40.50	44.50	62.00
Overall (*n* = 24)	30.00	39.50	49.50	47.92	56.25	68.00
**PDAC**	**Minimum**	**Q1**	**Q2**	**Average**	**Q3**	**Maximum**
Males (*n* = 11)	52.00	58.00	68.00	64.45	69.50	73.00
Females (*n* = 9)	49.00	67.00	68.00	65.78	71.00	75.00
Overall (*n* = 20)	49.00	60.00	68.00	65.05	70.25	75.00

**Table 9:** Descriptive of age depending on clinical group, benign cyst (OC), and ovarian cancer (OV).

Clinical Group	Minimum	Q1	Q2	Average	Q3	Maximum
OC (*n* = 25)	17.00	34.00	48.00	46.48	54.00	79.00
OV (*n* = 23)	16.00	48.00	59.00	60.09	78.00	82.00
Overall (*n* = 48)	16.00	37.75	51.00	53.00	73.00	82.00

## Data Availability

The data presented in this study are available on request from the corresponding author.

## Supplementary Materials

The following are available online at https://www.mdpi.com/article/10.3390/ijms22126501/s1, Table S1: Dynamic light scattering data, Table S2: Main descriptive indexes for the two fluorescence curves-associated parameters, in each clinical group (BD and PDAC), in the presence and absence of DHP-bMPA, Table S3: Univariate analysis of fluorescence curves-associated parameters of BD group and PDAC group in presence and absence of DHP-bMPA, Table S4: FS_score_ and FS_score_-DHP-bMPA values comparison between BD and PDAC, Table S5: Main descriptive indexes for the two fluorescence curves-associated parameters, in each clinical group, OC and OV, without and with DHP-bMPA, Table S6. Univariate analysis of parameters of OC and OV groups, Table S7. FS_score_ and FS_score_-DHP-bMPA values comparison between OC and OV.
