# Supplementary material for: Fluorescence Liquid Biopsy for Cancer Detection Is Improved by Using Cationic Dendronized Hyperbranched Polymer
Source: Int J Mol Sci. 2021 Jun 17;22(12):6501. doi: 10.3390/ijms22126501 (PMC8234380; doi:10.3390/ijms22126501)
Supplement: Supplementary file 1 [file ijms-22-06501-s001.zip › ijms-1209368-supplementary.pdf]

Supplementary Materials

Fluorescence Liquid Biopsy for Cancer Detection Is Improved by Using Cationic Dendronized Hyperbranched Polymer

Violeta Morcuende-Ventura [1,2,†], Sonia Hermoso-Durán [2,3,†], Natalia Abian-Franco [4], Roberto Pazo-Cid [5], Jorge L. Ojeda [6], Sonia Vega [2], Oscar Sanchez-Gracia [7], Adrian Velazquez-Campoy [2,3,8,9,10], Teresa Sierra [1,*] and Olga Abian [2,3,8,9,11,*]

Table S1. Particle-size determination according to dynamic light scattering measurements.

	Diameter (nm)	Zeta Potential (mV)
**DHP-bMPA (PBS)**	22 ± 4 nm	10.8 ± 0.4
**HS**	8 ± 1 nm	
**DHP-bMPA + HS**	36 ± 2 nm	

Table S2. Main descriptive indexes for the two fluorescence curves-associated parameters, in each clinical group (BD and PDAC), in the presence and absence of DHP-bMPA.

**DHP-bMPA**	**BD (n = 25)**	**Minimum**	**Q1**	**Q2**	**Average**	**Q3**	**Maximum**
Without	G1	1.03	1.06	1.07	1.07	1.09	1.13
	WL_{max}	443.00	462.00	462.00	459.88	462.00	464.00
With	G1	1.30	1.38	1.43	1.42	1.46	1.51
	WL_{max}	430.00	437.00	439.00	437.32	439.00	440.00
**DHP-bMPA**	**PDAC (n = 20)**	**Minimum**	**Q1**	**Q2**	**Average**	**Q3**	**Maximum**
Without	G1	1.04	1.07	1.10	1.12	1.15	1.27
	WL_{max}	441.00	443.00	444.00	450.40	460.50	463.00
With	G1	1.20	1.28	1.31	1.33	1.40	1.49
	WL_{max}	418.00	438.75	441.00	432.85	443.50	463.00

Notes: *G1 informs about the asymmetry of the spectrum and WLmax is the wavelenght at the maximum fluorescence signal.*

Table S3. Univariate analysis of fluorescence curves-associated parameters of BD group and PDAC group in presence and absence of DHP-bMPA.

DHP-bMPA	Parameter	BD (n = 25)	PDAC (n = 20)	*p*-Value
Without	G1	1.06 [1.06;1.09]	1.10 [1.07;1.15]	**0.019**
	WL_{max}	462.00 [462.00;462.00]	444.00 [443.00;460.50]	**0.001**
With	G1	1.43 [1.38;1.46]	1.31 [1.28;1.40]	**< 0.001**
	WL_{max}	439.00 [437.00;439.00]	438.50 [425.00;439.00]	0.379

Notes: *median [Q1;Q3]; p-Values were calculated according to Wilcoxon test. G1 informs about the asymmetry of the spectrum and WL_{max} is the wavelenght at the maximum fluorescence signal.*

Table S4. FS_{score} and FSscore- DHP-bMPA values comparison between BD and PDAC.

Model	BD (n = 25)	PDAC (n = 20)	*p*-Value
FS_{score}	0.25 [0.23;0.30]	0.66 [0.32;0.86]	**0.001**
FS_{score}- DHP-bMPA	0.13 [0.08;0.24]	0.86 [0.73;0.96]	**< 0.001**

Notes: *median [Q1;Q3]; p-Values were calculated according to Wilcoxon test.*

Table S5. Main descriptive indexes for the two fluorescence curves-associated parameters, in each clinical group, OC and OV, without and with DHP-bMPA.

DHP-bMPA	OC (n = 25)	Minimum	Q1	Q2	Average	Q3	Maximum
Without	G1	1.05	1.10	1.17	1.16	1.19	1.38
	WL_{max}	439.00	443.00	443.00	448.96	461.00	463.00
With	G1	1.09	1.22	1.26	1.27	1.32	1.50
	WL_{max}	418.00	440.00	441.50	439.68	444.50	465.00
**DHP-bMPA**	**OV (n = 24)**	**Minimum**	**Q1**	**Q2**	**Average**	**Q3**	**Maximum**
Without	G1	1.04	1.08	1.12	1.14	1.16	1.32
	WL_{max}	413.00	443.00	454.00	451.42	462.00	463.00
With	G1	1.38	1.45	1.50	1.52	1.56	1.68
	WL_{max}	424.00	431.00	434.50	434.29	439.00	441.00

Table S6. Univariate analysis of parameters of OC and OV groups.

DHP-bMPA	Parameter	OC (n = 25)	OV (n = 24)	*p*-Value
Without	G1	1.17 [1.10;1.19]	1.12 [1.08;1.16]	0.289
	WL_{max}	443.00 [443.00;461.00]	454.00 [443.00;462.00]	0.207
With	G1	1.27 (0.10)	1.52 (0.08)	**<0.001**
	WL_{max}	440.00 [439.00;441.00]	434.50 [431.00;439.00]	**<0.001**

Notes: *average (standard deviation) and median [Q1;Q3] are provided depending on the normality character of the parameter distribution; p-Values were calculated according to t-test or Wilcoxon test, depending on the normality character of the parameter distribution.*

Table S7. FSscore and FSscore- DHP-bMPA values comparison between OC and OV.

**Model**	**OC (n = 25)**	**OV (n = 24)**	***p*-Value**
FS_{score}	0.46 [0.45;0.55]	0.49 [0.46;0.55]	0.174
FS_{score}- DHP-bMPA	< 0.01 [< 0.01;0.05]	0.95 [0.90;0.99]	**< 0.001**

Notes: *median [Q1;Q3]; p-Values were calculated according to Wilcoxon test.*
